# Aquaporin 2 is differentially expressed in granulosa cells of various stages of human follicles and is regulated by luteinizing hormone

**DOI:** 10.3389/fcell.2025.1647476

**Published:** 2025-08-18

**Authors:** Zetong Zheng, Jifan Tan, Minghui Chen, Xiubing Zhang, Simin Liu, Yangxing Wen, Lingli Long, Canquan Zhou, Yubin Li

**Affiliations:** ^1^ Reproductive Medicine Center, The First Affiliated Hospital of Sun Yat-sen University, Guangzhou, Guangdong, China; ^2^ Guangdong Provincial Key Laboratory of Reproductive Medicine, The First Affiliated Hospital of Sun Yat-sen University, Guangzhou, Guangdong, China; ^3^ Reproductive Medicine Center, Shenzhen Maternity and Child Healthcare Hospital, Women and Children’s Medical Center, Southern Medical University, Shenzhen, Guangdong, China; ^4^ Clinical Trials Unit, The First Affiliated Hospital, Sun Yat-sen University, Guangzhou, Guangdong, China

**Keywords:** aquaporin 2, luteinized granulosa cells, follicular fluid, follicular growth, theca cells

## Abstract

**Introduction:**

Several aquaporins (AQPs) are involved in the influx of water to form follicular fluid, and AQP2 may play a crucial role in follicular growth. However, the specific roles of Aquaporin (AQP) 2 and AQP6 in granulosa cells (GCs) during follicular fluid (FF) formation, as well as their relationship with gonadotropins (Gn), remain unclear.

**Methods:**

Luteinized granulosa cells (LGCs) were isolated from follicles of different diameters. Western blot indicated that AQP2 protein levels in LGCs increased as follicles grew larger after luteinization. Immunohistochemistry of human ovarian sections showed that AQP2 levels decreased as follicles progressed from primordial to antral stages. Subsequently, isolated LGCs were treated with varying concentrations of follicle-stimulating hormone (FSH), luteinizing hormone (LH), and estradiol (E2); LH, but not FSH or E2, significantly elevated AQP2 expression. To dissect the underlying signaling pathways, LGCs were further cultured with LH, db-cAMP (a cAMP analog), or forskolin (an adenylate cyclase activator). H89 (a PKA inhibitor) or PD98059 (an ERK1/2 signaling inhibitor) was applied in the presence of LH to evaluate crosstalk between the Gn/cAMP and MAPK cascades.

**Results:**

AQP2 levels in LGCs increased with follicle enlargement after luteinization but decreased as follicles progressed from primordial to antral stages. LH, but not E2, significantly induced AQP2 expression in LGCs in a dose-dependent manner. Forskolin mimicked the stimulatory effect of LH on AQP2 expression. PD98059, but not H89, abolished LH-induced AQP2 up-regulation and inhibited ERK1/2 phosphorylation, indicating potential crosstalk between cAMP and MAPK signaling.

**Discussion:**

This study provides the first evidence for the mechanisms by which AQP2 influences follicular growth and FF formation, highlighting LH-driven, ERK1/2-dependent regulation of AQP2. These findings offer new insights into the ovarian microenvironment and identify potential therapeutic targets for follicle growth disorders.

## 1 Introduction

Gonadotropins (Gn), including follicle-stimulating hormone (FSH) and luteinizing hormone (LH), play essential roles in follicular development (Dewailly et al., 2016; Messinis et al., 2010; Oduwole et al., 2021). Based on their responsiveness to gonadotropins, follicles can be classified into three distinct phases: the Gn-independent phase (primordial, primary, and secondary follicles), the Gn-responsive phase (preantral and early antral follicles), and the Gn-dependent phase (antral follicles) (McGee and Hsueh, 2000; Orisaka et al., 2009). During these phases, Gn exerts different effects on follicular growth. In the early stages, FSH levels rise between cycles, creating the “FSH window,” which is responsible for cyclic recruitment and the selection of the dominant follicle (McGee and Hsueh, 2000). As follicles develop, they become less dependent on FSH and more dependent on LH (Mihm et al., 2006), with a certain threshold of LH required for follicular maturation (Franco et al., 2009; Jeppesen et al., 2012). The LH surge ultimately triggers follicular luteinization and the production of progesterone (Richards, 1994). When follicles enter the Gn-dependent phase and become antral, follicular fluid (FF) is produced to maintain the homeostasis of the follicular microenvironment, marked by the formation of the antrum (Orisaka et al., 2009; Hillier et al., 1994). This process is crucial for follicular development, as FF provides essential nutrients and growth factors to the oocyte. However, the exact mechanisms underlying FF formation remain largely unknown.

Aquaporins (AQPs) are a family of transmembrane proteins that facilitate the transport of water and small solutes across cell membranes. Within the female reproductive system, AQPs have emerged as critical regulators of fluid homeostasis with distinct isoforms demonstrating tissue-specific expression patterns and hormonal responsiveness (Nascimento et al., 2023; Thoroddsen et al., 2011). While all AQP subtypes except AQP10 have been detected throughout the female reproductive tract, their functional significance in ovarian physiology remains incompletely characterized.

Previous investigations have established important roles for AQPs in various reproductive tissues. For example, AQP1, -2, -3, -5, and -6 play roles in vaginal lubrication (Kim et al., 2011), AQP 1, -3, -8 in cervix (Shi et al., 2012) and AQP1, -2, -3 in uterus (Feng et al., 2008; Mobasheri et al., 2005). In the endometrium, AQP2 expression demonstrates menstrual cycle-dependent variation (He et al., 2006) and has been shown to be directly regulated by 17β-estradiol (Kim et al., 2009) through an identified estrogen response element in its promoter region (Zou et al., 2011). Similarly, AQP5 has been implicated in embryo implantation processes in rodent models, with its expression similarly modulated by estrogenic signaling (Kobayashi et al., 2006). These findings collectively underscore the intimate relationship between AQP-mediated fluid dynamics and steroid hormone action in reproductive tissues (Kordowitzki et al., 2020). Within the ovarian context, emerging evidence suggests that AQPs may play particularly crucial roles in follicular development and function. Multiple AQP isoforms, including AQP1, -2, -3, and -4, have been identified in human ovarian granulosa and theca cells (Thoroddsen et al., 2011), with their expression patterns potentially linked to follicular stage and gonadotropin sensitivity. Our previous work revealed stage-specific expression patterns of AQP2 and AQP6 in granulosa cells across follicular development (tong et al., 2023), suggesting their involvement in follicular fluid production and homeostasis (He et al., 2006; tong et al., 2023; Skowronski et al., 2019). The hormonal regulation of these AQPs is particularly noteworthy, with both FSH and LH demonstrating capacity to modulate their expression, potentially through both genomic and non-genomic mechanisms. Despite these advances, significant gaps remain in our understanding of AQP biology in human ovarian function. The precise mechanisms by which specific AQP isoforms contribute to follicular fluid formation, antral follicle expansion, and ultimately follicular rupture remain to be fully elucidated. Furthermore, the potential clinical relevance of AQP dysregulation in conditions such as polycystic ovary syndrome (Tian et al., 2018; Xiong et al., 2019) or ovarian hyperstimulation syndrome (Jin et al., 2012) warrants further investigation.

This study seeks to address several of these knowledge gaps through comprehensive characterization of AQP2 and AQP6 expression patterns across human follicular development stages and investigation of their regulation by gonadotropins *in vitro*. By elucidating these relationships, we aim to provide novel insights into the molecular mechanisms governing follicular fluid dynamics and their potential implications for ovarian physiology and pathophysiology.

## 2 Materials and methods

### 2.1 Patients

Our study included 26 women undergoing *in vitro* fertilization (IVF) and intracytoplasmic sperm injection (ICSI) at Reproductive Medicine Center of the First Affiliated Hospital of Sun Yat-sen University between March and September 2022. We included women aged 20–38 years, with a body mass index of 18–24 kg/m^2^, and no history of hereditary or familial diseases. All patients underwent the standard treatment of gonadotropin-releasing hormone antagonist (GnRH-ant) cycles (Chen et al., 2023) or gonadotropin-releasing hormone agonist (GnRH-a) cycles as indicated before (Liu et al., 2022).

The retrieved ovarian tissues were obtained from 14 patients at the Department of Gynecology of the First Affiliated Hospital of Sun Yat-sen University, who had undergone salpingo-oophorectomy with or without hysterectomy for a uterine or unilateral ovarian malignant tumor before chemotherapy or radiotherapy. All study methods were approved by the Ethics Committee of the First Affiliated Hospital of Sun Yat-sen University.

### 2.2 Human luteinizing GCs (LGCs) and theca cell (TCs) isolation, culture, and treatment

Cells isolated from follicular fluid during oocyte retrieval after controlled ovarian stimulation (COS) and hCG trigger. Although we acknowledge that not all GCs in antral follicles undergo complete luteinization before oocyte pick-up, the majority are exposed to luteinizing signals (hCG) in our clinical protocol. Thus, following precedents in literature (Wen et al., 2010; Lindeberg et al., 2007; Sperduti et al., 2021), we refer to these cells as luteinizing GCs (LGCs). For LGCs, the FF of each patient was collected in groups according to follicle diameter: small follicle group (S, <13 mm), medium follicle group (M, 13–18 mm), and large follicle group (L, >18 mm). FF from each group and per patient was pooled and centrifuged for 20–25 min at 1,065 × *g* to remove red blood cells. LGCs were purified, as previously described (Chen et al., 2016), using 50% Percoll (GE17-0891-01, Sigma, St. Louis, Missouri, United States) by gradient centrifugation for 10 min at 650 × g. The cells at the interface were removed, resuspended in phosphate-buffered saline (PBS, YJ0014, Yongjin, Guangdong, China), and centrifuged for 5 min at 450 *× g*. Ovarian tissue fragments were then removed from the LGCs suspension with a 40-μM cell strainer (CSS013040, Jet Bio, Guangdong, China). For TCs, all FF from one patient was collected, and TCs were purified as previously described (Chen et al., 2023). Briefly, FF was centrifuged for 10 min at 650 *× g*. The precipitate was reconstituted in PBS and filtered with a 100-μm strainer (CSS013100, Jet Bio). The follicle shells were repeatedly resuspended in PBS during filtration to remove the GCs. The pieces of follicle shells that remained on the filter membrane were collected and digested with 5 mg/mL collagenase I (C8140, Solarbio, Beijing, China) solution at 37°C for approximately 30 min and pipetted every 10 min to collect the dispersed cells. After being washed with PBS twice, and resuspended in PBS, cell suspensions were mixed and filtered with a 40-μm cell strainer to remove undigested tissue and centrifuged for 5 min at 650 *× g*.

Red bold cell lysis buffer (R1010, Solarbio) was added in purified target cells, plated on a 12-well plate, and cultured in Dulbecco’s modified Eagle medium/Ham’s F12 (C11330500BT, Gibco, Grand Island, NE, United States) supplemented with penicillin (100 U/mL), streptomycin (100 μg/mL) (15140122, Gibco, Grand Island, NE, United States), and 10% fetal bovine serum (FSP500, ExCell Bio, Jiangsu, China) in a 37°C incubator with 5% CO_2_. Cells were cultured for 1 day and exposed to 1.0 mIU/mL, 10 mIU/mL, and 100 mIU/mL FSH (Puregon®, NV Organon, Oss, Netherlands); 0.5 IU/mL, 1.0 IU/mL, and 1.5 IU/mL LH (Luveris®, MerckSerono, Geneva, Switzerland); 10^–9^ M, 10^–8^ M, and 10^–7^ M E2 (Sigma, St. Louis, Missouri, United States); 500 μM dibutyryl-cyclic-adenosine monophosphate (db-cAMP, HY-B0764, MCE); or 10 μM forskolin (FSK, HY-15371, MCE) for 24 h, respectively, and 10 μM H89 dihydrochloride (H89, HY-15979A, MCE) or 10 μM PD98059 (HY-12028, MCE) was added with 1.5 IU/mL LH for 10 min, 30 min, 60 min, and 120 min, respectively. Finally, cells were harvested, and stored (−80°C) for mRNA expression analysis and protein expression.

### 2.3 Western blot analysis

Total protein was isolated from LGCs or TCs using an ice-cold radioimmunoprecipitation assay (RIPA, EpiZyme) containing a protease inhibitor cocktail (GRF101, EpiZyme, Shanghai, China). Proteins were electrophoresed on SDS-PAGE gels (PG112, EpiZyme, Shanghai, China) and transferred onto Immobilon transfer membranes (Millipore, MA, United States). After being blocked in 5% skim milk for 1 h at room temperature, membranes were incubated with primary antibodies at 4°C overnight. After washing three times with TBS-T (TBS, with 0.1% Tween-20), the membranes were incubated with horseradish peroxidase-conjugated secondary antibodies against rabbit IgG for 1 h at room temperature. The primary antibodies used in this study were Beta-actin (20536-1-AP, Proteintech, Hubei, China), AQP2 (PA5-78808, Thermo Fisher Scientific, Waltham, MA, United States), AQP6 (PA5-103615, Thermo Fisher Scientific), ERK1/2 (11257-1-AP, Proteintech), and Phospho-ERK1/2 (28733-1-AP, Proteintech). The secondary antibodies used in this study were HRP-conjugated Affinipure Goat Anti-Rabbit IgG (H + L) (SA00001-2, Proteintech). The membranes were treated with an ECL reagent (SQ203, EpiZyme), and the blots were visualized using a iBright FL1500 Imaging System (Thermo Fisher Scientific, Waltham, MA, United States). ImageJ software (NIH, Bethesda, MD, United States) was used for quantification of band intensity. Results were normalized to those of β-actin (ACTB) and were presented as fold change to control groups (regarded as 1).

### 2.4 RNA isolation and real-time quantitative PCR (RT-qPCR)

Total RNA was isolated using RNAiso Plus (9109, Takara, Tokyo, Japan), according to the manufacturer’s instructions. HiScript III RT SuperMix for qPCR (+gDNA wiper) (R323. Vazyme, Jiangsu, China) was used for reverse transcription. Real-time PCR was performed using Taq Pro Universal SYBR qPCR Master Mix (Q712, Vazyme) on a QuantStudio 5 Real-Time PCR System (Thermo Fisher Scientific). Target gene Ct values were normalized to β-actin Ct values, and the average normalized target gene Ct values were calculated. Relative gene expression levels were compared with those of controls, and all RT-qPCRs were performed in triplicate. Primers used are listed in Supplementary Table S1.

### 2.5 Immunohistochemistry (IHC)

Ovarian tissues obtained from each patient were divided into 2-3 tissue blocks for paraffin embedding. From each tissue block, two representative slides were prepared for Aquaporin 2 (AQP2) and Aquaporin 6 (AQP6) immunohistochemical staining, respectively. Tissues were fixed in 4% formaldehyde, embedded in paraffin, and sectioned at 4 μm thickness following standard protocols. After deparaffinization and antigen retrieval, sections were blocked with normal goat serum and incubated overnight at 4°C with either rabbit anti-human AQP2 polyclonal antibody (1:300 dilution, PA5-78808, Thermo Fisher Scientific) or AQP6 polyclonal antibody (1:100 dilution, PA5-103615, Thermo Fisher Scientific). Following primary antibody incubation, slides were washed and incubated with horseradish peroxidase-labeled goat anti-rabbit secondary antibody for 50 min, followed by three 5-min washes with PBS. The peroxidase-antibody complex was visualized using 3,3′-diaminobenzidine (DA1010, Solarbio), and sections were counterstained with hematoxylin. Negative control experiments were performed by omitting the primary antibody.

The identification and classification of follicles were performed according to the number of granulosa cell layers and the presence of follicular antrum, as described in previous literature (Griffin et al., 2006). For quantitative analysis, primordial and primary follicles were rigorously counted only when the oocyte nucleus was clearly visible in the histological section. Secondary follicles were identified based on granulosa cell morphology regardless of oocyte nucleus visibility, while antral follicles were classified by the presence of distinct antral spaces. This conservative approach was adopted to mitigate potential quantification errors arising from oocyte loss during tissue processing or sectioning artifacts. To ensure precise evaluation of granulosa cell-specific staining, immunostaining quantification was performed only within explicitly demarcated granulosa cell areas (indicated by red dashed lines in Figures 2A,B), excluding oocytes and theca cell layers. Immunostaining of GCs was semi-quantitatively assessed using a combined scoring system (0-12 points) that incorporated both staining intensity (0 = no staining; 1 = weak; 2 = moderate; 3 = strong) and the percentage of positive cells (0 = 0%; 1 = 1–25%; 2 = 26–50%; 3 = 51–75%; 4 > 75%), as previously described (Ni et al., 2021).

### 2.6 Immunofluorescence (IF)

To evaluate AQP2 and AQP6 deposition and distribution, multiplex immunofluorescence was performed as previously described (Liu et al., 2022). Ovarian sections were dewaxed and treated with 0.01% (w/v) sodium citrate for 20 min for antigen retrieval.

Sections were permeabilized with PBS containing 0.5% Triton-X-100 (PBST) for 20 min and incubated for 1 h with 5% goat serum (AR1009, Boster Biological Technology, Hubei, China) at 25°C. The GCs were then probed with an AQP2 polyclonal antibody diluted to 1:300, or AQP6 polyclonal antibody diluted to 1:100 overnight at 4°C. The sections were sequentially incubated with Alexa Fluor 555-conjugated or 488-conjugated secondary antibody diluted 1:200 (ab150144, ab150081, Abcam, Cambridge, United Kingdom) for 1 h. The slides were counterstained with 4′,6-diamidino-2-pheynylindole (ab18804, Abcam). Digital images were acquired using a fluorescence microscope (Olympus, Japan).

### 2.7 Statistical analysis

All statistical analyses were performed using GraphPad Prism software. Data are presented as mean ± SEM, with P < 0.05 considered statistically significant. For Western blot analysis of AQP2 expression in granulosa cells, paired t-tests were appropriately employed for the following methodological considerations: (Dewailly et al., 2016): When comparing follicular stages within individual patients, all bands on the same membrane represented technical replicates from the same patient sample; (Messinis et al., 2010); For *in vitro* stimulation experiments, bands on each membrane contained pooled samples from the same cohort of patients, enabling within-membrane comparisons against matched controls. This paired analytical approach accounts for inter-patient variability while maintaining statistical power for detecting treatment effects.

## 3 Results

### 3.1 Expression of AQP2 and AQP6 in human LGCs from follicles with different diameters on oocyte pick-up (OPU) day

**FIGURE 1:**
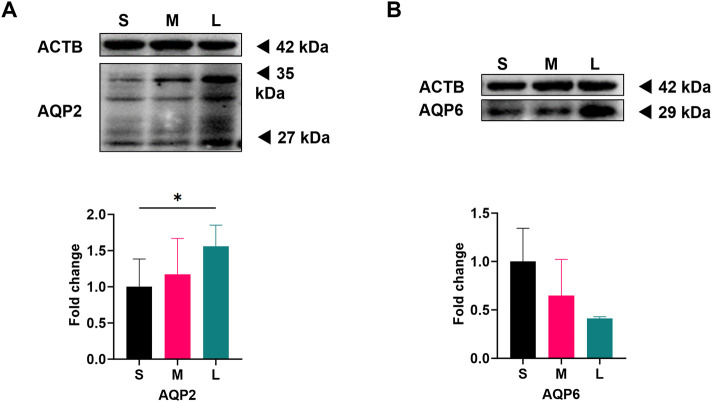
Expression of AQP2 and AQP6 in luteinized granulosa cells (LGCs) from different diameter follicles on oocyte pick-up (OPU) day. Follicles retrieved from one woman on OPU day were divided into three groups: small (S), medium (M), and large (L), and corresponding LGCs were purified from follicular fluid (FF). As individual heterogeneity, only when LGCs from the three groups were qualified, samples were delivered for analysis. Western blot results for Aquaporin 2 (AQP2, n = 3, **(A)** and Aquaporin 6 (AQP6, n = 3, **(B)** in S, M, and L groups of LGCs. ImageJ software was used to determine the band intensities. Results were normalized to those of β-actin (ACTB) and were presented as fold change to S groups (regarded as 1). The 27 kDa band represents the non-glycosylated form of AQP2, and the 35 kDa band for glycosylated form. Results are shown as the mean ± SEM.*P < 0.05. S, small follicle group; M, median follicle group; L, large follicle group; AQP, Aquaporin; ACTB, β-actin.

According to our previous study (tong et al., 2023), the mRNA expression of AQP2 in LGCs increased significantly, and, although without significant difference, the mRNA expression of AQP6 showed a downward trend with the increase of follicle diameter. Here, we further detected the protein level of these two AQP in LGCs. As previous described (tong et al., 2023), follicles were divided into three groups according to their diameter on OPU day under transvaginal ultrasound measurement: small, medium, and large follicle groups, and GCs were purified and collected. AQP2 expression levels in LGCs showed a positive correlation with follicular size, with increasing expression observed as follicles developed (Figure 1A). In contrast, our results showed the opposite trend in the expression of AQP6. Contrary to initial observations, quantitative analysis revealed reduced AQP6 expression in the large follicle group (L) compared to small follicles (S) after normalization across three independent replicates, though this difference lacked statistical significance (Figure 1B). Complete raw grayscale values and normalized quantifications are provided in Supplementary Table S2. Our Western blot quantification results demonstrate concordance between AQP protein expression trends and the mRNA level changes documented in prior studies (tong et al., 2023).

### 3.2 Expression of AQP2 and AQP6 in human GCs from antral follicles to primordial follicles

**FIGURE 2:**
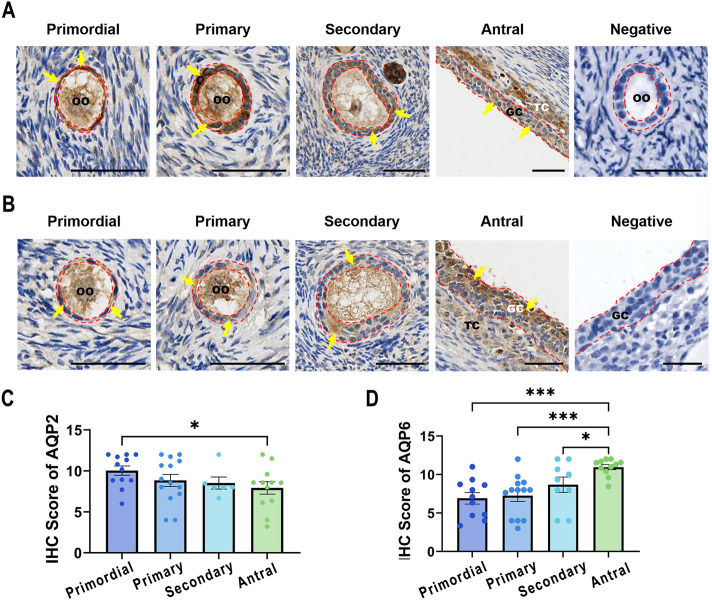
Expression of AQP2 and AQP6 in various phases of follicles. Follicles were retrieved from women undergoing ovariectomy and divided into four phases: primordial (AQP2: N = 12, n = 388; AQP6: N = 11, n = 371), primary (AQP2: N = 14, n = 199; AQP6: N = 13, n = 124), secondary (AQP2: N = 6, n = 16; AQP6: N = 9, n = 17), and antral (AQP2: N = 12, n = 281; AQP6: N = 11, n = 172) follicles. **(A,B)** Representative images of IHC staining for AQP2 **(A)** and AQP6 **(B)**. Yellow/brown staining indicates positive signals of the targeted proteins and all sections were lightly stained with hematoxylin (blue) for nuclear indicating. The red dashed line outlines the granulosa cell layer with positive signals indicated by arrowheads. Scale bar, 50 μm. GC, granulosa cells; TC, theca cells; OO, oocyte. **(C,D)** The intensity scores for Aquaporin 2 (AQP2, **(C)** and Aquaporin 6 (AQP6, **(D)** were measured using IHC in GCs from various phases of follicles. Results are shown as the means ± SEM. *P < 0.05, ***P < 0.001. N, patients size; n, follicle size.

To determine whether AQP2 and AQP6 play a role in follicle growth by producing FF, we characterized the expression of the two proteins in follicles in different phases (primordial, primary, secondary, and antral follicles) of normal ovaries using IHC. The expression of AQP2 and AQP6 in GCs at different follicular stages varied among patients. Mild staining of AQP2 was detected in GCs from secondary and antral follicles (Figures 2A,C). AQP2 staining was intense in the granulosa cell layer of primordial and primary follicles, and the expression level between primordial follicles and antral follicles was significantly different (the average IHC score of AQP2 for primordial and antral follicles was 10.03, and 7.92, respectively, *P* = 0.0377). However, the most abundant AQP6 staining was detected in antral follicles rather than in primordial follicles. As the follicles grew from primordial to antral follicles, AQP6 expression levels increased (Figure 2B), and the increase was statistically significant (Figure 2D). The co-localization analysis (Supplementary Figure S3) further excluded hematoxylin interference.

Interestingly, AQP2 and AQP6 staining were observed to be localized in oocytes as well as cells in the theca layer (OO and TC in Figures 2A,B). Additional expression patterns in cumulus cells are detailed in Supplementary Figure S4.

### 3.3 Influence of FSH, LH, and E2 on the expression of AQP2 and AQP6 in cultured human LGCs

**FIGURE 3:**
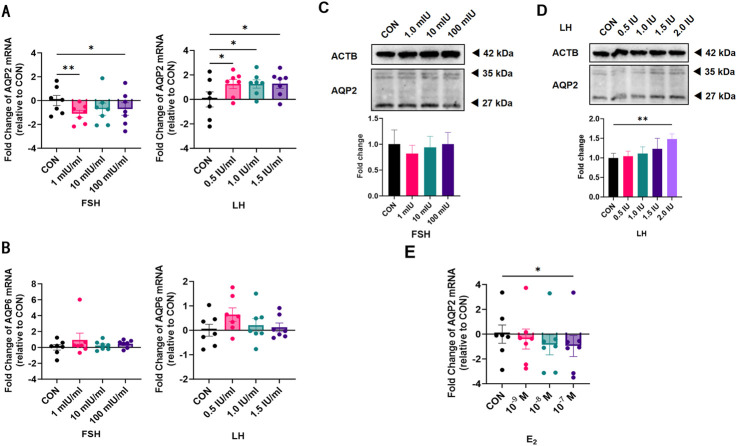
Effect of gonadotropin hormones on the expression of Aquaporin 2 (AQP2) and Aquaporin 6 (AQP6) in human luteinized granulosa cells (LGCs). LGCs purified from all follicle fluid regardless of follicle diameter obtained from artificial reproductive technology (ART) patients were cultured for 24 h and then treated with different concentrations of gonadotropin hormones. **(A,B)** The levels of mRNA for Aquaporin 2 (AQP2, **(A)**, n = 7) and Aquaporin 6 (AQP6, **(B)**, n = 7) were measured by quantitative PCR (qPCR), normalized to the levels of reduced glyceraldehyde-phosphate dehydrogenase (GAPDH) mRNA in each sample and were presented as fold change to the control group (CON, regarded as 1). **(C,D)** AQP2 was detected using western blots in LGCs cultured in different concentrations of follicle-stimulating hormone (FSH, **(C)**, n = 4) and luteinizing hormone (LH, **(D)**, n = 4). ImageJ software was used to determine the band intensities. Results were normalized to those of β-actin (ACTB) and were presented as fold change to the control group (CON, regarded as 1). **(E)** The level of mRNA for AQP2 was measured by qPCR, normalized to the levels of GAPDH mRNA (n = 7), and was presented as a fold change to the control group (CON, regarded as 1). Results are shown as the means ± SEM. *P < 0.05; **P < 0.01. CON, control group; AQP, Aquaporin; FSH, follicle-stimulating hormone; LH, luteinizing hormone; E2, estradiol.

To determine whether gonadotropins play a role in the expression of AQP2 and AQP6 in LGCs, LGCs were treated with FSH and LH for 24 h. LGCs expressed significantly lower levels of AQP2 in response to FSH while higher levels to LH as compared to the control (Figure 3A) in a dose-dependent manner. Similarly, AQP2 protein levels increased in LGCs treated with a relatively higher concentration of LH (Figure 3D), but not in the FSH treatment group (Figure 3C). Neither FSH nor LH influenced the expression of AQP6 in LGCs (Figure 3B).

To clarify whether the effect of LH on AQP2 was induced by E2, we measured AQP2 mRNA levels in LGCs treated with different concentrations of E2 for 24 h. A dose-dependent decreasing trend was observed in AQP2 mRNA expression level (Figure 3E).

### 3.4 Influence of db-cAMP, forskolin, H89, and PD98059 on the LH-induced expression of AQP2 in LGCs

**FIGURE 4:**
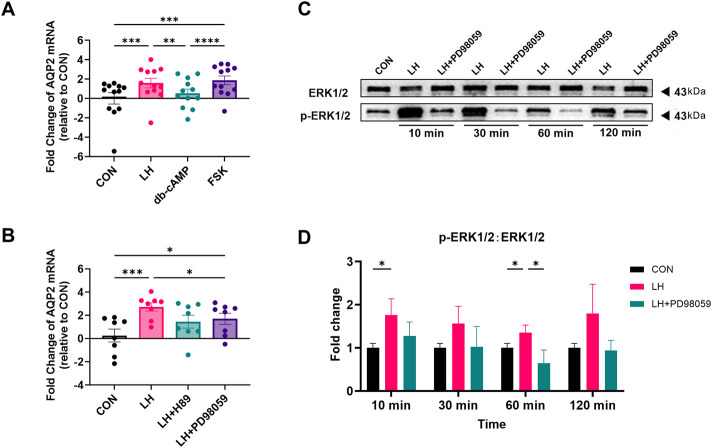
Effect of forskolin, db-cAMP, and PD98059 on the expression of Aquaporin 2 (AQP2) in cultured human luteinized granulosa cells (LGCs). **(A,B)** After being treated with 1.5 IU/mL luteinizing hormone (LH), 500 μΜ dibutyryl-cyclic-adenosine monophosphate (db-cAMP), 10 mΜ forskolin (FSK) (A, n = 12), 1.5 IU/mL LH with 10 μΜ H89 dihydrochloride (H89), or 10 μΜ PD98059 (B, n = 8), the level of mRNA for Aquaporin 2 (AQP2) was measured by quantitative PCR (qPCR), normalized to the levels of glyceraldehyde-3-phosphate dehydrogenase (GAPDH) mRNA in each sample, and were presented as fold change to the control group (CON, regarded as 1). **(C,D)** ERK1/2 and phospho-ERK1/2 (p-ERK1/2) were detected using western blots in LGCs treated with 1.5 IU/mL, with or without 10μΜ PD98059 at different times. ImageJ software was used to determine the band intensities. Results were normalized to those of ERK1/2 and were presented as fold change to control groups (CON, regarded as 1) (n = 4). Results are shown as the mean ± SEM. *P < 0.05; **P < 0.01; ***P < 0.001. AQP, Aquaporin; CON, control group; LH, luteinizing hormone; ERK, extracellular regulated kinase; pERK, phospho-ERK; db-cAMP, dibutyryl-cyclic-AMP; FSK, forskolin; H89, H89 dihydrochloride, inhibitor for protein kinase A (PKA); PD98059, selective MEK inhibitor and ERK1/2 inhibitor.

To determine whether the cyclic adenosine monophosphate/protein kinase A (cAMP/PKA) signal transduction pathway plays a role in the regulation of LH-induced AQP2 increase in LGCs, dibutyryl-cyclic-adenosine monophosphate (db-cAMP), a stabilized cyclic AMP (cAMP) analog, and forskolin (FSK), an inducer of intracellular cAMP formation, were added to the culture medium for 24 h. As shown in Figure 4A, FSK significantly increased the AQP2 mRNA level in LGCs, as LH did, but not db-cAMP.

H89 dihydrochloride (H89) acts as an inhibitor for PKA (Mihm et al., 2006; Ni et al., 2021) and was added to the culture medium with LH. As is shown in Figure 4B, H89 did not affect the increased mRNA level of AQP2 induced by LH in LGCs. To investigate the ERK1/2 pathway in LGCs, PD98059, an ERK1/2 signaling inhibitor, was used. Our results showed that PD98059 counteracted LH induction in LGCs (Figure 4B). The ratio of the phosphorylated ERK1/2 (PERK) to ERK1/2 (pERK1/2:ERK1/2) was found to be significantly increased with LH treatment, and was significantly decreased by PD98059 (Figures 4C,D).

### 3.5 Influence of FSH, LH, and E2 on expression of AQP2 in cultured human TCs

**FIGURE 5:**
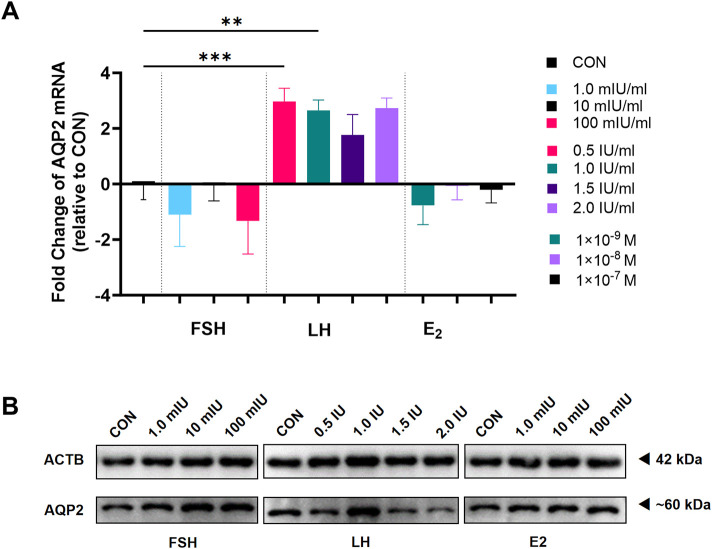
Effect of gonadotropin hormones on the expression of Aquaporin 2 (AQP2) in theca cells (TCs). TCs purified from all follicle fluid regardless of follicle diameter obtained from artificial reproductive technology (ART) patients were cultured for 24 h and then treated with different concentrations of follicle-stimulating hormone (FSH), luteinizing hormone (LH), and estradiol (E2). **(A)** The levels of mRNA for Aquaporin 2 (AQP2, n = 9) were measured by quantitative PCR and normalized to the levels of reduced glyceraldehyde-phosphate dehydrogenase (GAPDH) mRNA in each sample. **(B)** AQP2 was detected using western blots in TC cultured in different concentrations of FSH, LH, and E2. Results are shown as the mean ± SEM. **P < 0.01, ***P < 0.001. CON, control; AQP, Aquaporin; FSH, follicle-stimulating hormone; LH, luteinizing hormone; E2, estradiol.

As TCs had a high level of AQP2 expression in ovarian slides (Figure 2B), we gathered primary TCs and applied different levels of FSH, LH, and E2 to determine the role of these hormones in the expression AQP2 in TCs. As indicated in Figure 5, the level of AQP2 expression increased significantly after LH treatment, not only in mRNA level, but also protein level; however, neither FSH nor E2 influenced the expression of AQP2 in TCs. AQP2 in TCs presented as a glycosylated form (Kim et al., 2011) at approximately 60 kDa, apparently distinct from GCs.

## 4 Discussion

The formation of the follicle antrum marks the transition from preantral to antral follicles, after which the follicle antrum increases rapidly (Skowronski et al., 2019), with the largest volume of the human follicle reaching a size of approximately 20 mm in diameter. The rapid increase in follicular diameter is closely related to the increase in FF (He et al., 2006; Skowronski et al., 2019), and water influx is mainly transcellular and mediated by AQPs (Messinis et al., 2010). Animal experiments have shown that FF generation is related to AQP5, AQP7, AQP8, and AQP9 (Dewailly et al., 2016; Oduwole et al., 2021; Mihm et al., 2006; Hillier et al., 1980). In humans, AQP7 and AQP9 are present at relatively high levels in LGCs (Franco et al., 2009), and evidence shows that the significant increase in the follicle antrum during ovulation was related to AQP2 and AQP3 (Jeppesen et al., 2012). In our previous study, mRNA levels of AQP2 and AQP6 were found to be changed as follicular diameter increased, and the change was likely related to Gn. Herein, we further validate the changes in the protein levels of AQP2 and AQP6 and describe them in GCs from preantral to antral follicles and the regulation of gonadotropin-related changes.

We found that LGCs had higher protein levels of AQP2 and stable levels of AQP6 in follicles with larger diameters, which was consistent with the previous finding of mRNA expression. Gn plays a vital role in the development of ovarian follicles (Richards, 1994; Chen et al., 2023; Chen et al., 2016), while its regulation of AQPs at the aspect of FF formation is still unclear.

By using LGCs, we found that FSH as well as E2 inhibited AQP2 expression, while LH had an increasing effect, consistent with the results of Thoroddsen et al. (Thoroddsen et al., 2011). In their study, a marked increase in AQP2 mRNA levels during the early ovulatory phase (EO, 12–18 h after rhCG) was found, compared to the preovulatory phase (PO, the size of the dominant follicle ranged between 14 and 17.5 mm), which remained elevated throughout the ovulation period in the median levels of the late ovulatory (LO, 18–24 h after rhCG) and postovulatory (PSO, 44–77 h after rhCG) phases, respectively (Thoroddsen et al., 2011). In our experiment, LGCs were gathered at the time of oocyte extraction, which was approximately 36 h after the LH peak, as well as triggered ovulation. With the growth of follicles, follicles with FSH dependence gradually become more dependent on LH, which may explain the increased AQP2. Furthermore, after the LH peak, E2 decreases; that is, the inhibition of AQP2 is reduced, playing a synergistic role in promoting AQP2 expression with LH. Thus, our results suggest a role for AQP2 in the mechanisms leading to FF accumulation and follicular rupture.

The function of LH depends on the stage of follicular development (Liu et al., 2022) and is crucial for the transition from preantral to antral follicles and the success of ovulation (Kim et al., 2011; Ni et al., 2021; Gosden et al., 1988). LH binding to its receptors can initiate a G-protein-coupled signaling cascade that changes in gene expression in GCs (Hirshfield, 1991; McConnell et al., 2002). This also induces migration and cytoskeletal shape changes in isolated GCs (Lee et al., 2020; Yang et al., 2005) by mediating several pathways, such as the cyclic AMP-protein kinase A (cAMP/PKA) pathway (McConnell et al., 2002; Balasch and Fábregues, 2006; Lee et al., 2016), the ERK cascade (Jaffe and Egbert, 2017; Liu et al., 2014), AKT phosphorylation, JAK-STAT signaling pathway (Owen and Jaffe, 2024), and HIPPO pathway (Amsterdam et al., 2003; Godin et al., 2022). Notably, there is a crosstalk between Gn/cAMP and the MAPK signaling cascade (Lindeberg et al., 2007; Priyanka and Medhamurthy, 2007), which can be found in KGN, the granulosa cell line (Tani et al., 2004), and other cells (Kandaraki et al., 2018). In our study, forskolin increased AQP2 mRNA levels in GCs by activating PKA, as indicated in the principal cells of collecting tubules (Donadeu and Ascoli, 2005), and the increase was similar to that of LH. However, 500 μM db-cAMP displayed no effect on the AQP2 mRNA level, indicating that db-cAMP may not be suitable for investigation on AQP2 in GCs, or that a higher dosage should be explored. Interestingly, H89 did not influence AQP2 mRNA levels, whereas PD98059 antagonized the LH effect significantly, but not thoroughly. The obtained results indicated that there may be crosstalk between the cAMP and MAPK pathways, and LH may regulate AQP2 directly via the cAMP-ERK1/2 pathway. However, further experiments concerning Raf-1 and higher db-cAMP concentration are necessary.

In natural follicle growth, AQP2 decreased from primordial, primary, and secondary to antral follicles, as revealed by our IHC data. This trend was opposite to our *in vitro* LGCs experiments, but was not contrary to the speculated role of LH in AQP2 regulation. Follicles from primordial to secondary are present in the Gn-independent or Gn-responsive phase, when Gn is not necessary for follicular growth. As follicles begin to form follicular antrum, the TC layer is formed, which is a key event that occurs during the transition stage when follicular growth increases and a steroidogenic response to Gn occurs (Liu et al., 2022; Godin et al., 2022; Carvalho et al., 2003). According to the two-cell-two-gonadotropin theory (Tajima et al., 2003), TCs and GCs interact, and E2 synergistic regulation to AQP2 can be shown. However, the regulation of AQP2 before the Gn-dependent phase remains to be clarified.

As both the results of Jeppesen et al. (2012) and our IHC data showed abundant expression of AQP2 in the TC cell layer, we conducted brief experiments on TCs. We found a LH-related increase of AQP2, which is similar to GCs, implying the syntactic function of TCs and GCs during FF formation and follicular rapture; further experiments should be conducted to confirm the related pathways in TCs.

In our IHC findings regarding the high density of AQP2 in cumulus cells, cumulus and mural GCs have different transcriptomic profiles (Tani et al., 2004; Fan et al., 2009; Stork and Schmitt, 2002), especially in the distribution of LH receptors, mRNA coding for LH receptors and steroidogenic capabilities, and in mRNAs encoding cholesterol side-chain cleavage cytochrome P450 and cytochrome P450 aromatase (Latham et al., 1999). Cumulus expansion is essential to support the metabolic needs of oocytes and plays an important role in regulating meiotic arrest and resumption (Dumesic et al., 2015). The high density of AQP2 in cumulus cells (Supplementary Figure S4) and their LH-responsive pattern suggest a functional role in ovulation. However, due to the limited availability of intact cumulus-oocyte complexes in our specimens, current observations preclude robust statistical quantification. Future studies employing optimized isolation protocols (e.g., cumulus cell retrieval during ART procedures) are warranted to validate these findings at both protein and transcriptional levels, thereby elucidating AQP2’s precise role in cumulus cell physiology.

The current study represents the first comprehensive demonstration of AQP2 and AQP6 expression across human follicular stages, from primordial to antral follicles, as well as the first *in vitro* evaluation of GnRH agonist (Gn) effects on both AQPs. A key limitation involves potential differences between isolated luteinized GCs (LGCs) and physiologically intact GCs. Although minor contamination by non-luteinized GCs may theoretically exist in our LGC preparations, the standardized controlled ovarian stimulation (COS) protocol and hCG trigger in our study design suggest that follicular fluid-derived GCs are predominantly undergoing luteinization. Thus, we contend that any residual non-luteinized GCs constitute a negligible, functionally insignificant population that does not compromise inter-group comparability.

Methodologically, antral follicle classification by diameter alone carries inherent biological variability, preventing precise categorical divisions. Future studies employing *in vitro* maturation (IVM)-derived non-luteinized GCs could further validate our findings, while additional investigations remain essential to clarify AQP2’s mechanistic roles in follicular fluid formation and follicle rupture.

In summary, the present study demonstrates an LH-induced increase in AQP2 expression in human LGCs. Moreover, we revealed for the first time that AQP2 decreased, while AQP6 increased as follicles grew from primordial, primary, and secondary to antral follicles. This essential finding reveals a possible mechanism for FF formation during follicle growth. However, it remains to be determined whether the crosstalk between cAMP and ERK1/2 is responsible for LH-induced regulation. Future studies are needed to confirm the role of AQP2 in GC during the preantral follicle phase and in TC. Nonetheless, the present study provides further experimental evidence that AQP2 plays a significant role in the mechanisms that lead to FF accumulation and follicular rupture. The current novel findings provide fundamental information that can be valuable to understand FF and follicle growth.

## Data Availability

The original contributions presented in the study are included in the article/Supplementary Material, further inquiries can be directed to the corresponding authors.

## Abbreviations

ART, Artificial reproductive technology; FF, Follicular fluid; FSH, Follicle-stimulating hormone; GC, Granulosa cells; LGC, Luteinized granulosa cells; LH, Luteinizing hormone; OPU, Oocyte pick-up; PBS, Phosphate-buffered saline; TC, Theca cell.
