# Supplementary material for: Aquaporin 2 is differentially expressed in granulosa cells of various stages of human follicles and is regulated by luteinizing hormone
Source: Front Cell Dev Biol. 2025 Aug 18;13:1647476. doi: 10.3389/fcell.2025.1647476 (PMC12399404; doi:10.3389/fcell.2025.1647476)
Supplement: Supplementary file 1 [file Table1.docx]

Supplementary Table S1 Primer sequences (Forward and Reverse) used for real-time PCR analysis

| Gene | Forward primers | Reverse primers |
| --- | --- | --- |
| *AQP2* | AGCCGCTCTGCTCCATGAGATCAC | GGCGGAAACAGCACGTAGTTGTAG |
| *AQP6* | CTTAGTTTCCTGGGTCCCTCCTG | GAAGCCGTCTCCAGGCTCTCT |
| *β-actin* | ACTCTTCCAGCCTTCCTTCC | AGCACTGTGTTGGCGTACAG |
