# Supplementary figures and images for: Aquaporin 2 is differentially expressed in granulosa cells of various stages of human follicles and is regulated by luteinizing hormone
Source: Front Cell Dev Biol. 2025 Aug 18;13:1647476. doi: 10.3389/fcell.2025.1647476 (PMC12399404; doi:10.3389/fcell.2025.1647476)

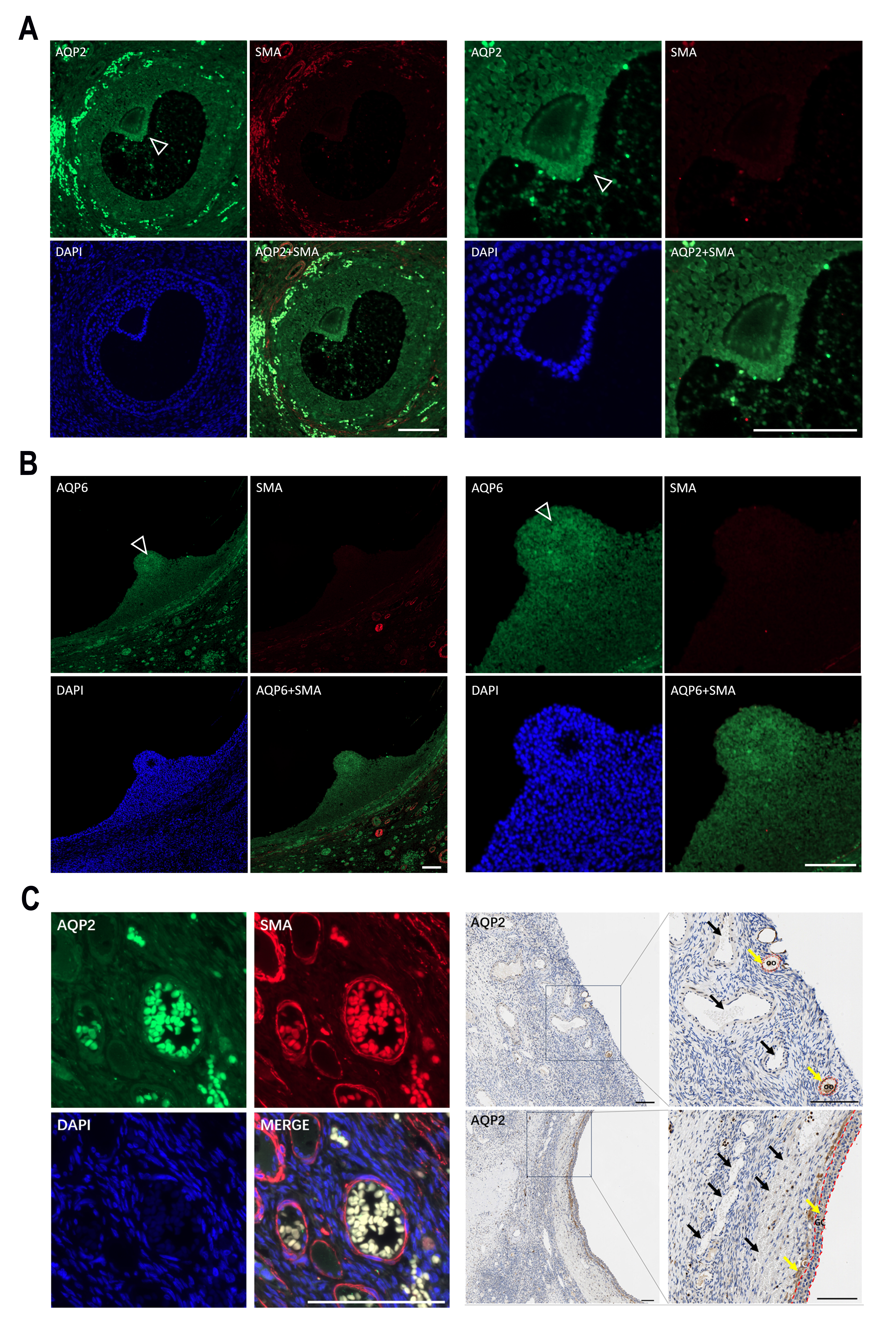

Supplement: Supplementary file 2 [file Image2.jpeg]

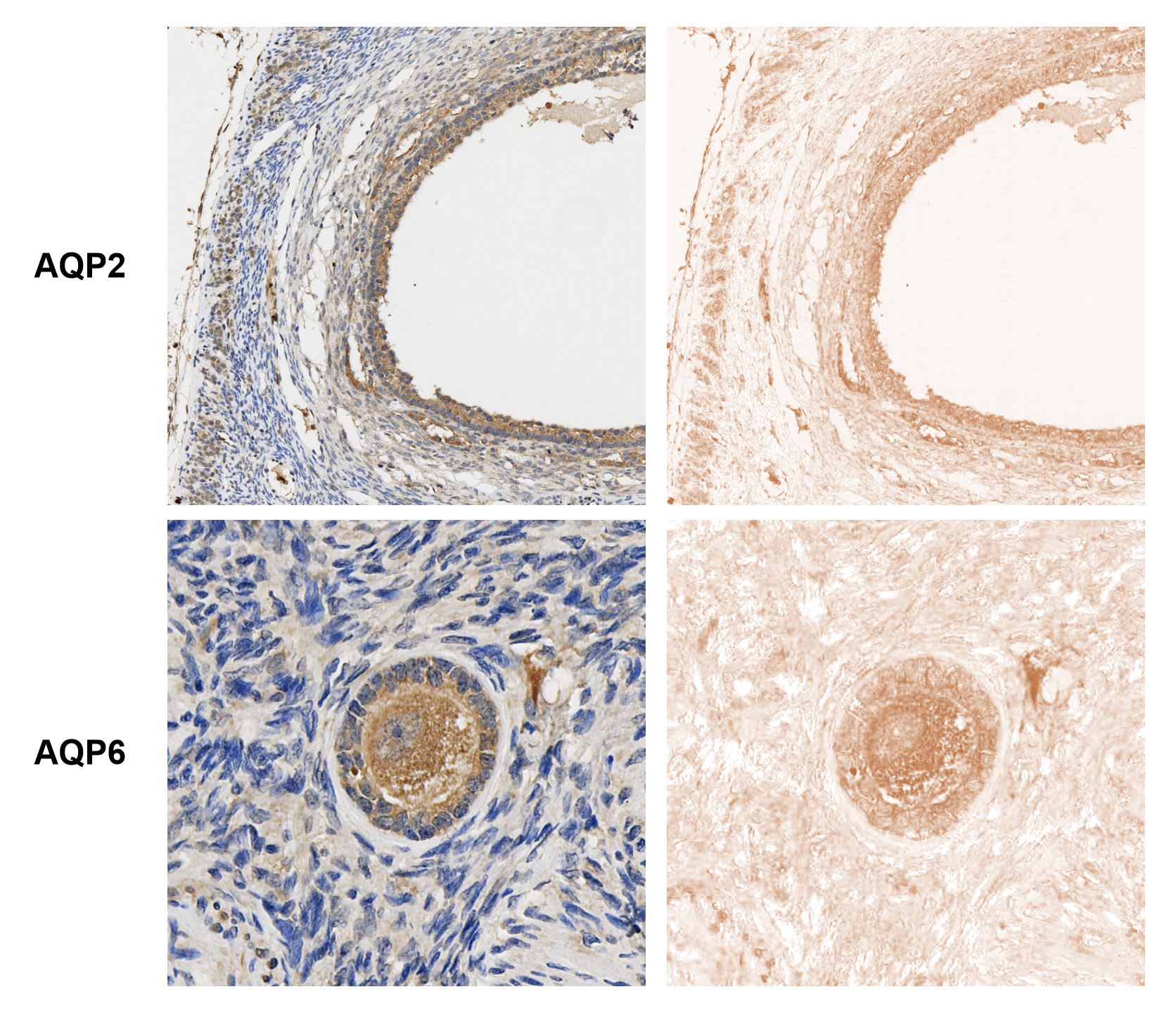

Supplement: Supplementary file 3 [file Image1.tif]
